# Population-Based Study of Bloodstream Infection Incidence and Mortality Rates, Finland, 2004–2018 

**DOI:** 10.3201/eid2710.204826

**Published:** 2021-10

**Authors:** Keiju S.K. Kontula, Kirsi Skogberg, Jukka Ollgren, Asko Järvinen, Outi Lyytikäinen

**Affiliations:** Helsinki University Hospital, Helsinki, Finland (K.S.K. Kontula, K. Skogberg, A. Järvinen);; Finnish Institute for Health and Welfare, Helsinki (J. Ollgren, O. Lyytikäinen)

**Keywords:** bloodstream infections, incidence, case-fatality rates, mortality rates, community-acquired infections, healthcare-associated infections, antimicrobial resistance, bacteria, fungi, MRSA and other staphylococci, streptococci, Finland

## Abstract

We evaluated the incidence, outcomes, and causative agents of bloodstream infections (BSI) in Finland during 2004–2018 by using data from the national registries. We identified a total of 173,715 BSIs; annual incidence increased from 150 to 309 cases/100,000 population. BSI incidence rose most sharply among persons >80 years of age. The 1-month case-fatality rate decreased from 13.0% to 12.6%, but the 1-month all-cause mortality rate rose from 20 to 39 deaths/100,000 population. BSIs caused by *Escherichia coli* increased from 26% to 30% of all BSIs. BSIs caused by multidrug-resistant microbes rose from 0.4% to 2.8%, mostly caused by extended-spectrum β-lactamase-producing *E. coli.* We observed an increase in community-acquired BSIs, from 67% to 78%. The proportion of patients with severe underlying conditions rose from 14% to 23%. Additional public health and healthcare prevention efforts are needed to curb the increasing trend in community-acquired BSIs and antimicrobial drug–resistant *E. coli*.

## Abstract 2

A 2-fold increase in incidence and death during this period emphasizes the need for additional prevention efforts.

Bloodstream infections (BSIs) are a major cause of illness and death worldwide. The incidence of BSIs has increased over time and reported BSI rates range from 122 to 220 cases/100,000 population (*1*–*7*). Rising incidence is probably related to an aging population and an increasing prevalence of underlying conditions and invasive procedures.

Despite advances in antimicrobial drug therapy, intensive care, and prevention strategies, BSIs cause an estimated 250,000 deaths annually in North America and Europe combined (*8*). Recent studies have reported BSI mortality rates of 21–32 deaths/100,000 population (*3*,*6*) and 1-month case-fatality rates of ≈17%–28% for nosocomial BSIs and 10%–19% for community-acquired BSIs (*9*–*12*). Higher mortality rates of 40%–50% have been observed in surveys of patients with BSIs or septic shock in hospital intensive care units (*13*,*14*). However, only limited population-based data are available concerning the incidence and outcome of BSIs (*1*,*3*,*4*,*6*,*10*,*12*,*15*). Most other reports mainly focus on selected hospitals or hospital units, a specific causative agent, or either healthcare-associated or community-acquired BSIs, and thus those studies represent select patient populations.

We used data from the national, laboratory-based surveillance system in Finland to analyze the annual incidence, causative agents, and outcomes of all BSIs in the country during 2004–2018. Our objective was to assess the burden and temporal trends of BSIs in Finland and identify targets for preventive interventions.

## Materials and Methods

### Study Setting and Population

Finland had a population 5.2 million in 2004 and 5.5 million in 2018. The country’s healthcare system is organized into 20 healthcare districts; there are 5 tertiary care hospitals and 15 secondary care hospitals, and the number of primary care hospitals varies by district. All clinical microbiology laboratories in Finland report all bacterial and fungal isolates from blood samples to the National Infectious Disease Register (NIDR) (*16*). These notifications are sent electronically and comprise specimen date; type of microbe; and the patient’s date of birth, sex, place of residence, and national identity code, a unique number given to each resident in Finland. NIDR merges multiple notifications of the same microbe from the same national identity code, indicating samples came from same person, and creates a single case if notifications occur within 3 months of each other.

**Figure 1:**
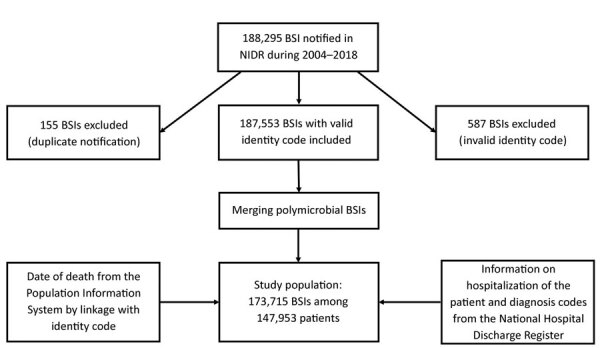
Flowchart of the data reviewed for inclusion in a study of the incidence of BSIs, Finland, 2004–2018. BSI, bloodstream infections; NIDR, National Infectious Disease Register.

In this retrospective study, we used NIDR data to identify all BSIs in Finland during 2004–2018. We included 187,553 BSIs with valid national identity codes in the study; we excluded 155 duplicate notifications, that is, those with same specimen date, microbe, and identity code (Figure 1). We retrieved date of death from the Population Information System (https://dvv.fi/en/population-information-system) by linking the patient’s national identity code with database information. We obtained information on patient hospitalization, including origin of the infection, and current and prior (1 year) diagnosis codes by linking to the National Hospital Discharge Register (HILMO).

The Ethics Committee of the Finnish Institute for Health and Welfare granted permission to analyze and link data from the NIDR and HILMO (approval no. THL/1349/6.02.00/2019). Because the data were already anonymized, patient informed consent was waived.

### Definitions

We defined the presence of BSI as occurrence of viable bacteria or fungi in bloodstream evidenced by positive blood cultures. We defined polymicrobial BSI as isolation of >1 bacterial or fungal species in blood cultures collected within 2 days (*16*).

We classified BSI as healthcare-associated when the first blood culture was obtained >2 days after admission to hospital or within 2 days after discharge (*17*). We also classified cases as healthcare-associated for patients who came from another healthcare facility. We classified cases as community-acquired when patients had no prior hospitalization and blood culture specimens were collected <2 days after hospital admission.

We defined underlying illness by using a validated algorithm for the Charlson comorbidity index (CCI) based on the International Classification of Diseases, 10th Revision (*18*,*19*). We scored underlying illness on 3 levels: low (CCI score of 0) for patients with no reported underlying diseases, medium (CCI score 1–2), or high (CCI score >3) (*10*). We defined all-cause mortality and case-fatality as death of a particular patient <30 days after the first positive blood culture. 

We defined the following bacteria as multidrug-resistant (MDR) microbes: extended-spectrum β-lactamase-producing (ESBL) *Escherichia coli* and *Klebsiella pneumoniae*, methicillin-resistant *Staphylococcus aureus* (MRSA), vancomycin-resistant *Enterococcus* (VRE) and carbapenemase-producing Enterobacteriaceae (CPE). We defined ESBL–*E. coli* and ESBL–*K. pneumoniae* as resistant or intermediately susceptible to third-generation cephalosporins. We defined CPE as *E. coli*, *K. pneumoniae*, and *Enterobacter* sp. resistant or intermediately susceptible to carbapenems.

### Analyses and Statistics

We used population data from Statistics Finland (https://www.stat.fi/index_en.html) as denominators to calculate age- and sex-specific BSI incidence and mortality rates. We determined average annual incidence and mortality rates according to the total number of BSI episodes, BSI deaths, and population during 2004–2018. We applied a Poisson regression model, or negative binomial regression model in case of overdispersion, to compare the observed trends in annual incidence and mortality rates and used a log-linear binomial regression model for case-fatality proportions. We analyzed the data by using SPSS Statistics 25 (IBM, https://www.ibm.com) and Stata 16 (StataCorp LLC, https://www.stata.com).

## Results

During 2004−2018, we identified a total of 173,715 BSIs among 147,953 patients in the NIDR (Figure 1). Median age among BSI patients was 70 (range 0–110) years; 52% were male and 48% female. Among all BSIs, 7,568 (4.4%) occurred in children <16 years of age, including 3,734 BSIs in infants <1 year of age. The average annual incidence was 216 BSI episodes/100,000 population and was higher among male (228 episodes/100,000 population) than female (203 episodes/100,000 population) patients. Overall BSI incidence was highest among patients >60 years of age (618 cases/100,000 population) and patients <1 year of age (431 cases/100,000 population). Among infants <1 year and persons >40 years of age, BSI incidence rates were higher in male than in female patients; only among persons 20–29 years of age was BSI incidence higher in female patients.

**Figure 2:**
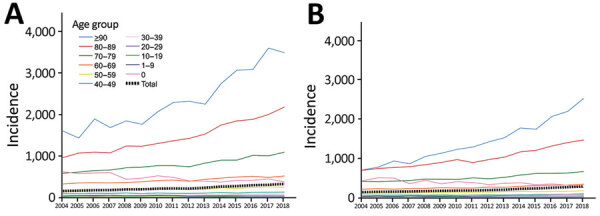
Annual incidence (cases/100,000 population) of bloodstream infections, by sex and age group, Finland, 2004–2018. A) Male patients; B) female patients.

During 2004–2018, the annual BSI incidence rose from 150 to 309 cases/100,000 population, an average annual increase of 5.2% (95% CI 4.8%–5.5%; p<0.01). BSI incidence increased in both sexes; among male persons, incidence increased from 155 to 327 cases/100,000 population, an average annual increase of 5.3% (95% CI 4.9%–5.7%; p<0.01); among female persons, incidence increased from 145 to 291 cases/100,000 population, an average annual increase of 5.0% (95% CI 4.6%–5.4%; p<0.01) (Figure 2). The increase in the annual incidence was most prominent among persons >90 years of age, from 1,155 to 3,005 cases/100,000 population, an average annual increase of 8.6% (95% CI 8.0%–9.1%; p<0.01). We observed a decreasing incidence only among infants <1 year of age, from 528 to 317 cases/100,000 population, an average annual decrease of 3.3% (p<0.01). We also noted a decreasing incidence in children <10 years of age, from 37 to 28 cases/100,000 population, an average annual decrease of 4.0% (p<0.01).

**Figure 3:**
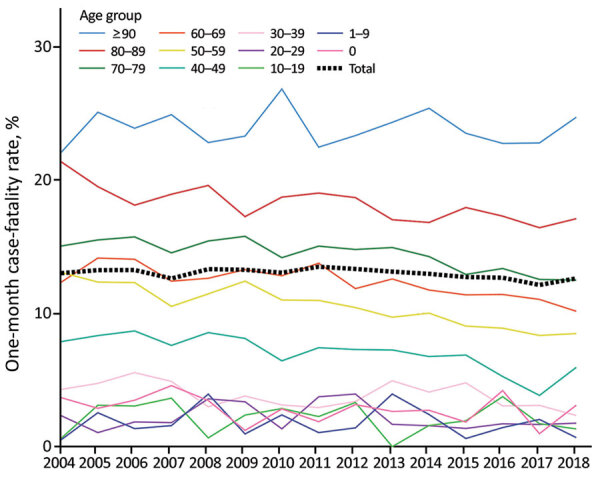
Annual 1-month case-fatality rates for bloodstream infections, by age group, Finland, 2004–2018.

**Figure 4:**
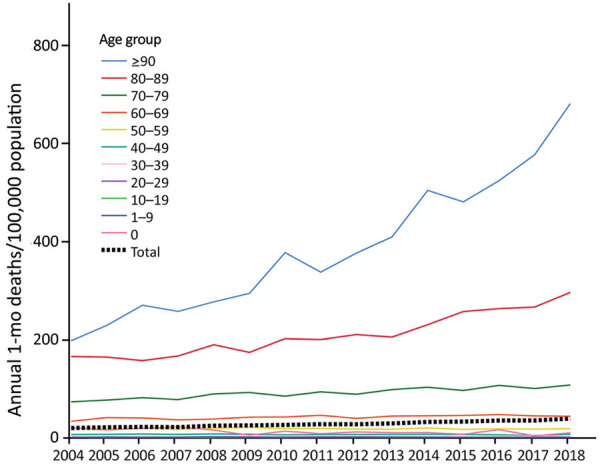
Annual average 1-month bloodstream infection deaths per 100,000 population, by age group, Finland, 2004–2018.

Among all reported BSI cases, 22,474 (13%) were fatal within 1 month; case-fatality rate was higher among male (13.7%) than female (12.1%) patients (relative risk 1.14, 95% CI 1.11–1.17; p<0.01). During 2004–2018, we noted a minor decrease in the 1-month case-fatality rate, from 13.0% to 12.6%, an average annual relative reduction of 0.4% (95% CI 0.1%–0.7%; p<0.01) (Figure 3). Among children and adolescents 1–19 years of age and among persons >90 years of age, the case-fatality rate increased slightly, but in other age groups we observed a descending rate. The average annual BSI mortality rate was 28 deaths/100,000 population during the study period. The mortality rate was higher for male patients in all age groups; among persons >20 years of age, mortality rates were >1.5-fold higher among male than female patients. The mortality rate increased with age; among persons >70 years of age, the rate was 148 deaths/100,000 population. The annual BSI mortality rate rose from 20 to 39 deaths/100,000 population during 2004–2018, and the overall average annual increase was 4.8% (95% CI 4.5%–5.1%; p<0.01) (Figure 4); the increase was 4.5% (p<0.01) among male patients and 5.2% (p<0.01) among female patients. The increase in mortality rate was most notable among persons >90 years of age, an average increase of 8.1% (p<0.01).

Among all BSIs, gram-positive bacteria caused 46% of infections, gram-negative bacteria 46%, fungi 1.5%, and other unclassified bacteria 0.2%. Polymicrobial BSIs accounted for 7% of all BSIs. *E. coli* was the most common causative pathogen (29% of all BSIs), but other identified pathogens included *S. aureus* (13%), coagulase-negative staphylococcus (CNS) (8%), β-hemolytic streptococci (8%), *Streptococcus pneumoniae* (7%), *Klebsiella* sp. (5%), and enterococci (4%). Gram-positive bacteria were the most common cause of BSIs in male patients (52% vs. 40% for female patients), whereas gram-negative bacteria were more prevalent in female patients (53% vs. 39% for male patients). Polymicrobial BSIs were more frequently noted in male patients than in female patients (7.4% vs. 5.6%), as were BSIs caused by fungi (1.7% vs. 1.2%). Altogether, 3,150 (1.8%) BSIs were caused by 3,168 MDR microbes; 2,503 (1.4%) BSIs were caused by ESBL–*E. coli* or ESBL–*K. pneumoniae*, 562 (0.3%) by MRSA, 66 (0.04%) by VRE, and 37 (0.02%) by CPE. Among 18 BSIs, 2 different MDR microbes were identified.

**Figure 5:**
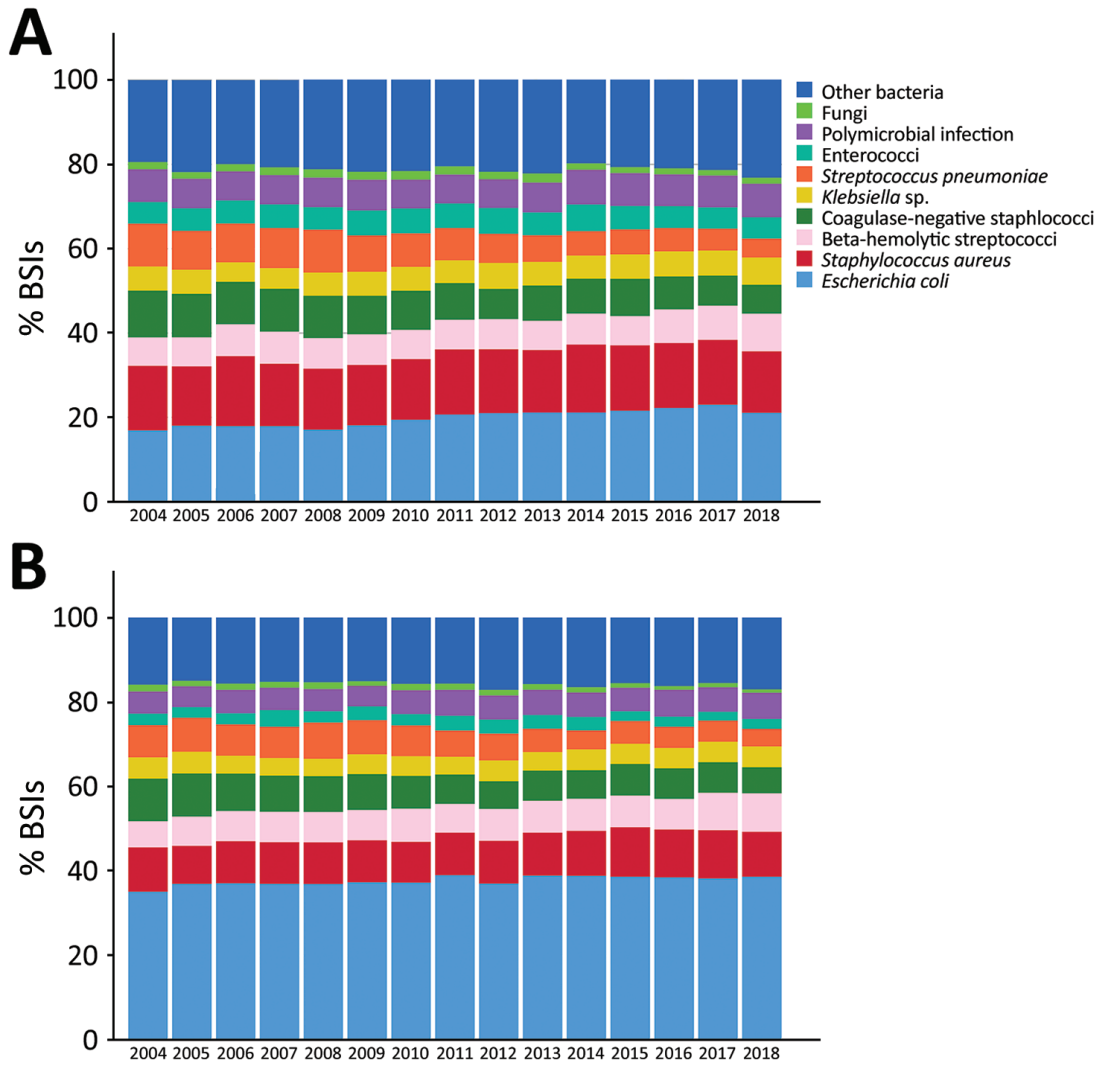
Frequency distribution of the most common causative agents of BSIs, by sex, Finland, 2004–2018. A) Male patients; B) female patients. BSI, bloodstream infections.

During 2004–2018, the proportion of BSIs caused by gram-negative bacteria rose from 42% to 48%, whereas BSIs caused by gram-positive bacteria decreased from 50% to 43% (Figure 5). Polymicrobial BSIs increased slightly from 6.5% to 7.1%, and BSIs caused by fungi decreased from 1.7% to 1.1% (Figure 5). Among the most common pathogens, the proportion of *E. coli* BSIs rose from 26% to 30%, whereas no change was noted in *S. aureus* BSIs (13%), and we observed a decline in BSIs caused by CNS (from 11% to 7%) and *S. pneumoniae* (9% to 4%). *Candida albicans* was the most common fungus, causing 0.9% of all BSIs and 63% of fungal BSIs, but the proportion of fungemia caused by other *Candida* species increased from 34% to 47%.

The annual incidence of *E. coli*, *S. aureus*, β*-*hemolytic streptococci, and *Klebsiella* BSIs rose >2-fold. In particular, *E. coli* BSIs rose from 39 to 91 cases/100,000 population and *S. aureus* from 19 to 39 cases/100,000 population. We noted a similar increase in the incidence of *E. coli* and *S. aureus* BSIs in both genders and the most prominent increase occurred among persons >80 years of age. The incidence of *S. pneumoniae* BSIs rose during 2004–2008, from 13 cases/100,000 population to 17 cases/100,000 population, and then decreased to 13 cases/100,000 population in 2018. We observed this decline in all age groups, but we noted the steepest reduction among persons <30 years of age, including infants <1 year of age.

The proportion of BSIs caused by MDR microbes rose from 0.4% in 2004 to 2.8% in 2018, mostly because of the increase in ESBL–*E. coli* BSIs, from 0 to 7% of all *E. coli* BSIs, an increase from 0 to 10% among male patients and from 0 to 6% in female patients. On the other hand, MRSA BSIs decreased from 3.1% to 2.0% of all *S. aureus* BSIs, and the annual number of MRSA BSIs ranged from 27 to 49 during 2004–2018. MDR microbes were causative agents in more BSIs leading to death within 30 days compared with other BSIs (2.4% vs. 1.7%).

Of the 173,715 BSI cases, 123,232 (71%) were community-acquired and 50,483 (29%) were healthcare-associated. During 2004–2018, the proportion of community-acquired BSIs rose from 67% to 78%, whereas healthcare-associated BSIs declined from 33% to 22%. The median CCI of all BSI patients was 1 (range 0–15); 38% had a low score (CCI 0) and 21% had a high score (CCI >3). During 2004–2018, the proportion of patients with a high CCI increased from 14% to 23%, but the proportion of patients with a low CCI decreased from 45% to 35%.

## Discussion

In our nationwide population-based study, BSI incidence and mortality rates increased 2-fold during 2004–2018 with the sharpest rise among persons ≥80 years of age. Community-acquired BSIs contributed to the rising incidence more than healthcare-associated BSIs did. The 1-month case fatality rate was 13% and remained rather stable over time despite the growing proportion of patients with high CCI scores. We noted a considerable, >2-fold, increase in the incidence of *E. coli* BSIs. The proportion of BSIs caused by MDR microbes was low, but we observed an ascending trend, mainly because of the increase in ESBL–*E. coli* BSIs.

During 2004–2018, the annual incidence of BSIs in Finland rose from 150 to 309 cases/100,000 population with an average annual rate of 216 BSI episodes/100,000 population. In 2 previous nationwide studies from Finland based on the same laboratory surveillance data, the average annual incidence rates were considerably lower than in our study, 125 cases/100,000 population during 1995–2002 and 159 cases/100,000 population during 2004–2007 (*3*,*20*). Similar increasing incidence rates have also been noted in other population-based surveys from Europe; from 114 to 166 cases/100,000 person-years during 1992–2006 in northern Denmark (*10*) and from 190 to 257 cases/100,000 person-years during 2002–2013 in mid-Norway (*6*). One recent population-based study, a report from Funen County, Denmark, during 2000–2008, demonstrated a decreasing overall incidence of BSIs (*15*).

We observed a marked, nearly 2-fold increase in all-cause mortality during 2004–2018; however, at the same time, the 1-month case-fatality rate decreased slightly, which might reflect advances in treatment for BSIs. A study from Norway during 2002–2013 demonstrated a similar mortality rate (32 cases/100,000 population) as in our study (28 cases/100,000 population) and showed higher rates in male than in female patients, comparable to our results (*6*). In that study, case-fatality rates decreased from 17.2% to 13.1% between 2002–2007 and 2008–2013 concurrent with an increasing incidence of BSIs and rising rates of blood culture sampling (*6*). A higher 30-day mortality rate was observed among hospitalized patients with bacteremia in Denmark, but that study also noted decreasing rates from 22.7% to 20.6% between 1992–1996 and 2002–2006 (*10*). A recent study from Sweden during 2000–2013 showed a 1-month case-fatality rate of 12.8%, similar to our results (*12*).

In our study, the proportion of BSI patients with a low CCI declined during 2004–2018 from 45% to 35%, but the proportion of those with a high CCI increased from 14% to 23%. Similarly, in a report from Denmark during 1992–2006, the proportion of BSI patients with a low CCI decreased from 42% to 33%, and the proportion of those with a high CCI rose from 20% to 30% (*10*). Furthermore, in a survey of BSIs from a county in Sweden, the proportion of patients with >1 underlying condition increased from 21% to 55% during 2000–2013 (*12*).

The average annual incidence of BSIs in our study was highest among older persons, as demonstrated in previous studies (*1*–*4*,*6*,*21*). In addition, we noted that the increase in the incidence over time was most notable among the oldest persons, those >90 years of age. Researchers widely recognize that the risk for BSIs increases as the population ages and as the life expectancy rises in industrialized countries. It is likely that the considerable rise in BSI incidence over time is also associated with increasing prevalence of underlying conditions, advanced treatments of chronic diseases, and implementation of invasive procedures. We noted a higher average annual BSI incidence among male patients, which aligns with results from previous reports (*1*–*3*,*6*,*21*), and is presumably related to higher prevalence of chronic diseases and predisposing factors among male persons.

In our study, healthcare-associated BSIs accounted for 29% of all BSIs and community-acquired BSIs for 71%; the proportion of healthcare-associated BSIs decreased during 2004–2018, but community-acquired BSIs increased. Similarly, a study in Sweden noted that hospital-acquired BSIs accounted for 33% and community-onset BSIs for 67% of all BSIs (*12*). In a survey from Denmark that reported 3 categories of BSIs by origin, the portion of nosocomial and community-acquired BSIs declined during 1992–2006, but healthcare-associated BSIs increased by >2-fold during the same time (*10*). The 2-day timeframe for our definition of healthcare-associated BSI was rather strict and might have led to underestimation of these BSIs. Some healthcare-associated BSIs possibly were inaccurately interpreted as community-acquired because the data on the origin of the infection were obtained from HILMO. The HILMO hospital discharge registry does not cover long-term care facilities, does not include information on possible outpatient invasive procedures before the BSI, and does not provide data on regular patient hospital visits for chronic hemodialysis or chemotherapy.

*E. coli* and *S. aureus* were the most common causative pathogens of all BSIs in our study, and in similar proportions as have been reported from Europe and North America (*1*,*3*,*12*,*15*,*21*). Our findings show the proportion of *E. coli* BSIs increased from 26% to 30% during 2004–2018, but no change was noted in *S. aureus* BSIs (13%). Similarly, in a report of BSIs in England during 2004–2008, the proportion of *E. coli* BSIs rose from 19% to 23%, but *S. aureus* BSIs decreased from 17% to 12% because of reduction in methicillin-resistant strains (*2*). A considerable increase in the proportion of *E. coli* BSIs also has been observed in Sweden (*12*), and in 2 other reports from countries in Scandinavia, the prevalence of bacteremia with urinary tract foci increased concurrently with rising rates of *E. coli* BSIs (*6*,*10*). In our study, the annual incidence of both *E. coli* and *S. aureus* rose by >2-fold during 2004–2018, which is comparable to results from previous population-based surveys (*22*,*23*). On the contrary, we noted that the incidence of *S. pneumoniae* BSIs decreased after 2008, which most likely is associated with the implementation of pneumococcal vaccines in Finland (*24*). This finding is in line with studies from Norway during 2002–2013 and England during 2004–2008 showing reduction in the incidence of *S. pneumoniae* BSIs after introduction of the conjugate vaccine to the childhood immunization schedule in 2006 (*2*,*6*).

In our study, the proportion of BSIs caused by MDR microbes was low (1.8%), but we observed a distinct ascending trend during 2004–2018; ESBL–*E. coli* BSIs increased the most. On the other hand, the proportion of MRSA BSIs decreased over time. In Finland, as in other Nordic countries, the proportion of MDR BSIs is typically low (*6*,*25*,*26*), whereas North America and most of Europe have considerably higher proportions of MDR BSIs, as shown in previous surveillance studies (*27*–*29*). These surveys also demonstrate a rising trend in MDR BSIs over time, as noted in our results.

Our study’s first limitations was that we did not have exact numbers of blood cultures performed during the study period. However, in a previous report from Finland the annual national blood culturing rate increased by one third during 1995–2002, from 2,752 to 3,667 cultures/100,000 population (*30*). Also, estimates from the Finnish Hospital Infection Program suggest that the median number of blood cultures among hospitalized patients in Finland increased by 25% during 2014–2018, from ≈120 to 150 blood culture sets/1,000 patient-days (*28*; O. Lyytikäinen, unpub. data). Recent reports from other countries have shown that increasing blood culture rates might influence the rising BSI incidence (*6*,*7*). Thus, higher culturing rates lead to improved detection of milder BSIs, which might contribute to the slightly decreasing case-fatality rates noted in our study. The rising incidence of BSIs also might reflect changes in the healthcare delivery system, such as centralized healthcare services in which patients with acute infections are treated at hospital emergency departments instead of in community healthcare centers, and blood cultures possibly are taken before patient conditions progress to severe BSI or when patients have milder infections and milder symptoms. Second, because the study was based on surveillance data, we did not have information on the focus of the infection, on possible delays in recognition and treatment of the infection, nor data on the appropriateness of antimicrobial therapy, which might have affected the outcome of BSIs. Third, we did not have information on the main cause of death of the patients, but presumably BSI was a contributing factor. Finally, we did not have data on patients’ underlying medical conditions other than those included in the CCI, nor did we have information on possible do-not-resuscitate orders for patients, which likely have influenced patient outcome, as we observed in our previous population-based case series of BSIs leading to early death (*31*).

Our population-based study of >170,000 BSIs in Finland during 15 consecutive years offers a comprehensive assessment of temporal trends and outcome of BSIs. We noted a 2-fold rise in the incidence and BSI mortality rates during 2004–2018. The proportion of BSIs caused by resistant microbes, mostly by ESBL–*E. coli*, rose over time, which could complicate antimicrobial therapy in the future and increase the risk for fatal BSI outcomes. Further research is required to assess the possible predisposing factors for BSI mortality. Overall, issues related to the increasing BSI incidence and death raised in our study ought to be evaluated separately in cases of community-acquired and healthcare-associated BSI in the future. Nonetheless, our data serve as a valuable point of reference for industrialized countries when estimating the effects of changes in the epidemiology of BSIs among an aging population and increasing antimicrobial resistance. Continuous BSI surveillance is needed to compose local recommendations for empiric antimicrobial treatment. Our findings underscore the necessity for substantial BSI prevention efforts and increased public and healthcare system awareness of severe infections. 
